# Understanding biocatalyst inhibition by carboxylic acids

**DOI:** 10.3389/fmicb.2013.00272

**Published:** 2013-09-03

**Authors:** Laura R. Jarboe, Liam A. Royce, Ping Liu

**Affiliations:** ^1^Department of Chemical and Biological Engineering, Iowa State UniversityAmes, IA, USA; ^2^Department of Microbiology, Iowa State UniversityAmes, IA, USA

**Keywords:** tolerance, membrane damage, transporters, acid resistance, intracellular pH, biocatalyst robustness, carboxylic acid toxicity

## Abstract

Carboxylic acids are an attractive biorenewable chemical in terms of their flexibility and usage as precursors for a variety of industrial chemicals. It has been demonstrated that such carboxylic acids can be fermentatively produced using engineered microbes, such as *Escherichia coli* and *Saccharomyces cerevisiae*. However, like many other attractive biorenewable fuels and chemicals, carboxylic acids become inhibitory to these microbes at concentrations below the desired yield and titer. In fact, their potency as microbial inhibitors is highlighted by the fact that many of these carboxylic acids are routinely used as food preservatives. This review highlights the current knowledge regarding the impact that saturated, straight-chain carboxylic acids, such as hexanoic, octanoic, decanoic, and lauric acids can have on *E. coli* and *S. cerevisiae*, with the goal of identifying metabolic engineering strategies to increase robustness. Key effects of these carboxylic acids include damage to the cell membrane and a decrease of the microbial internal pH. Certain changes in cell membrane properties, such as composition, fluidity, integrity, and hydrophobicity, and intracellular pH are often associated with increased tolerance. The availability of appropriate exporters, such as Pdr12, can also increase tolerance. The effect on metabolic processes, such as maintaining appropriate respiratory function, regulation of Lrp activity and inhibition of production of key metabolites such as methionine, are also considered. Understanding the mechanisms of biocatalyst inhibition by these desirable products can aid in the engineering of robust strains with improved industrial performance.

## INTRODUCTION

Carboxylic acids are useful biorenewable chemicals that can serve as precursors for drop-in replacements for petroleum-derived industrial chemicals (Mäki-Arvela et al., 2007; Lennen et al., 2010; Shanks, 2010; Carlos Serrano-Ruiz et al., 2012) and biologically-produced polymers (Wang et al., 2011) and alcohols (Perez et al., 2013). Much progress has been made in recent years in engineering workhorse biocatalysts, such as *Escherichia coli* and *S. cerevisiae*, for production of carboxylic acids (Lennen et al., 2010; Ranganathan et al., 2012; Zhang et al., 2012a,b), including recent reviews for both of these species (Abbott et al., 2009; Lennen and Pfleger, 2012; Liu and Jarboe, 2012).

However, in addition to our renewed interest in carboxylic acids as biorenewable chemicals, we have had a long history of using these compounds as food preservatives and soaps (Russell, 1991; Ricke, 2003; Kabara and Marshall, 2010). An even more ancient history involves the production of these compounds in the human digestive tract (Cummings and Macfarlane, 1991) and on human skin (Desbois and Smith, 2010). This functionality of carboxylic acids in inhibiting microbial activity presents a challenge in the microbial production of these compounds at a sufficiently high concentration and titer to enable an economically viable process.

Microbial inhibition by products or substrates is a relatively common problem in the production of biorenewable fuels and chemicals (Dunlop et al., 2011; Jarboe et al., 2011). This inhibitory action by carboxylic acids has already been cited as limiting performance in both *E. coli* (Lennen et al., 2011) and the cyanobacterium *Synechococcus elongatus* (Ruffing and Jones, 2012). The fact that *E. coli* strains have been developed that can produce 118 g/L (1.3 M) lactic acid and 83 g/L (0.70 M) succinic acid in defined minimal media demonstrates that organic acid tolerance can be increased by this organism (Jarboe et al., 2010). However, these two projects were enabled by the fact that production of the lactic and succinic acids were required to maintain redox and ATP balance, respectively, providing a selective marker for directed evolution (Jarboe et al., 2010). This is not possible in all cases and thus other strategies for increasing robustness are desired. It should be noted that titers of 0.78 M lactic acid have also been achieved in *S. cerevisiae* (Rossi et al., 2010). Information about the mechanism of inhibition can provide guidance to metabolic engineering strategies that increase microbial robustness (Dunlop et al., 2011; Jarboe et al., 2011; Wang et al., 2013) and thus enable a more economically viable and industrially relevant process (Chotani et al., 2000).

Here we provide a review of the current knowledge of the mechanisms of microbial inhibition by carboxylic acids. Since we are mainly interested in metabolic engineering for carboxylic acid production, we focus on *E. coli* and *S. cerevisiae*. Additionally, we mainly focus on straight-chain, saturated carboxylic acids of at least six carbons in length, such as hexanoic/caproic (C6:0), octanoic/caprylic (C8:0), decanoic/capric (C10:0), dodecanoic/lauric (C12:0), tetradecanoic/myristic (C14:0) and hexadecanoic/palmitic (C16:0) acids. Given its abundance in biomass hydrolysate, acetic acid (C2:0) has been the focus of extensive research (Mills et al., 2009) and is described here only when there is demonstrated relevance to or lack of data for longer-chain acids. There have been several excellent previously reviews on carboxylic acids tolerance (Ricke, 2003; Desbois and Smith, 2010; Kabara and Marshall, 2010), and this work is intended to serve only as an overview of specific concepts and to provide insight for future studies, not a comprehensive review of all relevant studies.

## CHARACTERIZATION OF INHIBITION AS A FUNCTION OF MOLECULE STRUCTURE AND MEDIA pH

The degree of inhibition by carboxylic acids can vary according to molecule identity (Kabara et al., 1972; Marounek et al., 2003; Kabara and Marshall, 2010), organism identity (Kabara et al., 1972), strain (DiezGonzalez and Russell, 1997) and growth condition (Viegas and Sa-Correia, 1995; Kasemets et al., 2006) and thus inhibitory concentrations are not listed here. A comparative study of inhibition by a weak acid (sorbic acid), an uncoupler (2,4-dinitrophenol) and a carboxylic acid (decanoic acid) observed that the carboxylic acid caused rapid cell death relative to the other two inhibitors and concluded that the mechanism of inhibition by carboxylic acids must be distinct from the other two molecule types (Stratford and Anslow, 1996). Desbois and Smith (2010) briefly review the association of molecule structure and shape with its potency as an inhibitor, but since most of these relationships deal with unsaturated molecules they are not discussed here. Our own studies have shown a significant increase in toxicity to *S. cerevisiae* on a molar basis as chain length increases from 6 to 8 to 10 carbons (Liu et al., 2013b), but this strong dependence on chain length was not observed with *E. coli* (Royce et al., 2013). The difference in the octanoic and decanoic responses in *S. cerevisiae* was also noted by a transcriptome-based study (Legras et al., 2010).

It is clear from the literature that carboxylic acid toxicity increases at lower pH values, particularly as the media pH nears the molecule pKa (Stratford and Anslow, 1996; Liu et al., 2013b; Royce et al., 2013). Another conserved factor of carboxylic acid toxicity is the link between toxicity and hydrophobicity (Zaldivar and Ingram, 1999), similar to the trends reported for solvent toxicity (Ramos et al., 2002). This relationship between toxicity, pKa and molecule hydrophobicity relates to transport of these molecules into the cell, as described in the following sections.

## MOVEMENT IN AND OUT OF THE CELL

We are more interested in systems that produce carboxylic acids than those that are challenged by exogenously-supplied carboxylic acids. However, understanding the toxicity of these compounds first requires an understanding of how they enter the cell; the bulk of the currently-available data on this topic relates to exogenously supplied carboxylic acids. Carboxylic acids can pass through the cell membrane via diffusion or a transporter (Nikaido, 2003).

The distribution of carboxylic acids between their protonated (HA) and ionic forms (H^+^ and A^-^) is a function of the system pH and the molecule’s pKa, as described by the Henderson–Hasselbalch equation

(1) $$pH\,\;=\;pKa\,\;+\;\log\frac{\left[A^{-}\right]}{\left[HA\right]}$$

It has been shown that for model membranes the limiting step for membrane permeation is also a function of carboxylic acid chain length (Evtodienko et al., 1996). Specifically, for chain lengths of 2–6 carbons, transport through the membrane is limited by diffusion of the anion when the external pH is below the molecule pKa, but limited by diffusion of the neutral form when the pH is greater than the pKa (Evtodienko et al., 1996). For longer-chain carboxylic acids, transport through these model membranes is limited by the diffusion of the anionic form at all pH values (Evtodienko et al., 1996). Changes in membrane properties that increase tolerance to carboxylic acids are discussed below.

Generally speaking, diffusion of carboxylic acids follow Overton’s Rule that membrane permeability is a function of molecule hydrophobicity (Al-Awqati, 1999; Kamp and Hamilton, 2006). Membrane permeability of the non-ionic form (*P^m^*) was measured for a variety of monocarboxylic acids and related to the more readily-available hexadecane/water partition coefficient (*K_p_*) with a correlation coefficient of 0.996 (Walter and Gutknecht, 1984) as

(2) $$\log(P^{m})\;=\;0.90\,\;\log(K_{p})\;+\;0.89$$

Accumulation of the anions within the cell has been asserted as one of the main mechanisms of microbial inhibition by carboxylic acids (Carpenter and Broadbent, 2009). The magnitude of this accumulation is a function of the external anion concentration and external pH (Carpenter and Broadbent, 2009); the biological implication of this accumulation is discussed below. Carpenter and Broadbent’s (2009) conclusions are consistent with Evtodienko’s et al. (1996) results for carbon chains of six carbons of less. The reason for the differences regarding longer-chain molecules is not clear.

There has been more success in identification of carboxylic acid transporters in *S. cerevisiae* than in *E. coli*. The Pdr12 ABC transporter was originally discovered during a study of sorbic (2,4-hexadienoic) acid toxicity and was shown to contribute to organic acid tolerance through the energy-dependent removal of carboxylate anions from the cell interior (Piper et al., 1998). Presumably Pdr12 is the transporter proposed to be necessary for acquisition of octanoic acid tolerance in other studies (Cabral et al., 2001), as it has since been shown to contribute to octanoic acid tolerance in *S. cerevisiae*, along with the Tpo1p transporter (Legras et al., 2010). The transporter-encoding *AQR1* gene has been shown to provide protection of *S. cerevisiae* against carboxylic acids of six carbons or less, but does not provide protection against octanoic acid (Tenreiro et al., 2002). Given the recent successes in increasing biocatalyst performance by provision of the appropriate product exporter (Dunlop et al., 2011; Park et al., 2011), this is an area that could possibly benefit from increased attention.

## MEMBRANE DAMAGE

Permeability of the cell membrane to carboxylic acids is indicative of the solubility of these compounds in this vital structure. The damage caused to the cell membrane has been presented as one of the main mechanisms of microbial inhibition by carboxylic acids (Ricke, 2003; Desbois and Smith, 2010). A recent nanoscale imaging study of membrane disruption by antimicrobial peptides was able to visualize formation of membrane pores and their expansion to the point of membrane disintegration (Rakowska et al., 2013), though it is not yet clear whether carboxylic acid membrane damage proceeds in this manner.

A recent omics-wide study of an *E. coli* strain engineered to produce a mixture of C8–C14 carboxylic acids to a total titer of approximately 300 mg/L concluded that membrane stresses are one of the major challenges faced by this strain (Lennen et al., 2011). Membrane stress was evidenced by increased permeability of the inner membrane to a nucleic acid dye and an 85% decrease in cell viability associated with carboxylic acid production, where cell viability was quantified by colony forming units relative to the non-producing strain in the same condition (Lennen et al., 2011). It was also noted that membrane damage associated with carboxylic acid production was increased relative to challenge with exogenously-supplied carboxylic acids (Lennen et al., 2011).

A transcriptional study of the conserved weak organic acid response in *S. cerevisiae* during anaerobic growth concluded that many of the genes activated in response to benzoate, sorbate, acetate, and propionate are related to cell wall structure and organization (Abbott et al., 2007). Our own transcriptome analysis of exogenous challenge of *S. cerevisae* with 43 mg/L (0.30 mM) octanoic acid at pH 5.0 and 30°C also concluded that membrane damage was the most significant effect (Liu et al., 2013b). Further studies were performed using Mg^2+^ as a representative small molecule that should be retained within the cell, but leaks out of damaged cell membranes. Mg^2+^ leakage was observed to increase in a dose-dependent manner in response to exogenously supplied C8 and in an increasing response to chain length when challenged with 0.30 mM hexanoic, octanoic or decanoic acids (Liu et al., 2013b). We detected no change in membrane fluidity or hydrophobicity (Liu et al., 2013b). A short period of adaptation to octanoic acid resulted in increased resistance to membrane damage, as evidenced by decreased Mg^2+^ efflux. Note that the importance of maintaining appropriate membrane fluidity and methods for its characterization have been reviewed elsewhere (Marguet et al., 2006). The mechanisms of this adaptation and the accompanying changes in membrane lipid composition are discussed below.

In *E. coli*, challenge with octanoic acid in minimal media at pH 7.0 and 37°C resulted in both a significant decrease of membrane polarization, indicative of an increase in fluidity, and Mg^2+^ leakage at levels approximately 50% of those observed with chloroform treatment (Royce et al., 2013). However, after a short period of adaptation to octanoic acid, cells became resistant to its fluidizing effect but not the membrane damage evidenced by Mg^2+^ efflux. During this adaptation, the membrane lipid composition changed, as discussed below, and the cell surface hydrophobicity significantly decreased (Royce et al., 2013). Consistent with this data, our analysis of a carboxylic acid-producing strain showed that membrane leakage, but not fluidity, increased as the carboxylic acid titer increased (Royce et al., 2013). This strain produced predominantly tetradecanoic and palmitic acids (Ranganathan et al., 2012) to a final titer of 600 mg/L and was characterized in minimal media at 30°C.

It should be noted that when Zaldivar and Ingram (1999) tested the sensitivity of *E. coli* to various organic acids, including hexanoic acid, well above concentrations that inhibits growth by 80%, only moderate amounts of Mg^2+^ leakage were detected and the authors concluded that membrane damage was not a key component of organic acid toxicity (Zaldivar and Ingram, 1999). This difference could possibly be due to the fact that these authors were studying an ethanol-producing *E. coli* strain in rich media.

This damage to the cell membrane can not only impact retention of valuable metabolites, such as Mg^2+^, but can also impact membrane-associated cell functions. Systems with damaged membranes frequently show evidence of oxidative stress, possibly due to decreased function of the electron transport chain (Lennen et al., 2011; Segura et al., 2012). Recent studies and reviews of carboxylic acid toxicity in *S. cerevisiae* have noted the link between toxicity and production of reactive oxygen species (ROS; Abbott et al., 2009; Legras et al., 2010). A thorough black box metabolic characterization was performed regarding octanoic acid toxicity with a strain of *E. coli* engineered to produce octanoic acid from octane (Rothen et al., 1998). It was shown that pulses of octanoic acid during growth in defined media resulted in transient decreases in production of CO_2_ and biomass, decreased utilization of glucose and O_2_ and increased production of acetate (Rothen et al., 1998), leading this author to propose that this is evidence of decreased aerobic respiration, possibly due to damage of the membrane-associated electron transport chain.

## CHANGES IN MEMBRANE PROPERTIES TO INCREASE TOLERANCE

As described above, a short period of adaptation to carboxylic acids can enable changes that increase tolerance to these inhibitory compounds. Understanding these changes can enable metabolic engineering strategies for increased tolerance. The extensive knowledge regarding membrane-related solvent toxicity may also be of use here (Ramos et al., 2002; Segura et al., 2012).

For example, it has been shown that mutant strains with decreased cell surface hydrophobicity have increased organic solvent tolerance (Aono and Kobayashi, 1997). This decrease in hydrophobicity was attributed to an increase in lipopolysaccharide content; lipopolysaccharide amino acid composition was unchanged (Aono and Kobayashi, 1997). Our own studies have shown that *E. coli *cell surface hydrophobicity decreases during adaptation to octanoic acid (Royce et al., 2013). Solvent tolerance is also frequently attributed to changes in the saturated/unsaturated ratio, *cis*/*trans* isomerization, the length of acyl chains, phospholipid head groups, lipopolysaccharide composition and membrane hydrophobicity (Ramos et al., 2002; Segura et al., 2012). Understanding the genetic and molecular mechanisms of these changes and their role in increasing tolerance can guide engineering efforts.

The most frequently-noted changes in response to carboxylic acid challenge, either exogenously supplied or during production, deal with the composition of the membrane lipids. For example, Lennen et al., 2011 study of an *E. coli* strain that produces free fatty acids noted an increase in the long-chain unsaturated fatty acid content in the cell membrane. Our own studies of *E. coli* MG1655 following 3 h of adaptation to octanoic acid at pH 7.0 showed a significant increase in average lipid length and a significant decrease in the saturated/unsaturated ratio (Royce et al., 2013). Our studies of octanoic adaptation of *S. cerevisiae* at pH 5.0 showed a similar, significant increase in average lipid length (Liu et al., 2013b). The saturated/unsaturated ratio was not as clear, showing a significant increase at moderate inhibitory octanoic acid concentrations of 43 and 72 mg/L (0.30 and 0.50 mM), but no significant difference between the control samples and those adapted to 115 mg/L octanoic acid (0.8 mM), a concentration which decreases the specific growth rate by more than 90% (Liu et al., 2013b).

The question remains as to whether this change in membrane composition is a microbial strategy for increasing carboxylic acid tolerance or a side effect of the presence of carboxylic acid and thus its own mechanism of inhibition. Lennen and Pfleger (2013) hypothesized that the decreased saturated fatty acid content was a mechanism of carboxylic acid toxicity and engineered their carboxylic acid-producing *E. coli* strain in order to restore the saturated fatty acid content to normal levels. Their engineering strategy was successful in increasing the saturated fatty acid content during carboxylic acid production, though levels were still higher than that observed for the non-producing control strain. In support of their hypothesis, viability of the carboxylic-acid producing strains was significantly increased in the strain engineered for increased saturated fatty acid content (Lennen and Pfleger, 2013). Similar results were observed when saturated fatty acid content was increased in order to increase *E. coli* ethanol tolerance (Luo et al., 2009). These results suggest that the presence of carboxylic acids precludes *E. coli* from maintaining the appropriate amount of saturated fatty acids in the cell membrane, leading to decreased viability.

Contrastingly, our research team interpreted the association between increased oleic acid (C18:1) content in the *S. cerevisiae* cell membrane after short-term adaptation to octanoic acid and increased resistance to membrane damage and growth inhibition by octanoic acid as evidence that increasing the oleic acid content in the membrane is beneficial for carboxylic acid tolerance (Liu et al., 2013b). We found that supplementing the growth media with 1.0 mM oleic acid increased the C18:1 content in the membrane to 54% (by area), relative to the 22% observed in the control cells and 35% in the cells adapted to 0.5 mM octanoic acid. This increased oleic acid content was accompanied by a significant decrease in octanoic acid-mediated Mg^2^^+^ leakage and decreased growth inhibition by 0.5 and 1.0 mM octanoic acid (Liu et al., 2013b). Subsequent metabolic engineering efforts were successful in increasing the oleic acid content independent of media supplementation, but not to the level needed for increased robustness (Liu et al., 2013b). Thus, at this point it is not clear whether there can be a general conclusion about either increasing or decreasing saturated fatty acid content as a means of increasing carboxylic acid robustness.

In addition to consistent reports of increased unsaturated fatty acid content in *E. coli* during carboxylic acid challenge or production, there have been consistent reports of increased cyclopropane fatty acid content (Lennen and Pfleger, 2013; Royce et al., 2013). The most common cyclopropane fatty acid in *E. coli* is C17:1, also refered to as C17cyc, produced by methylation of C16:1 phospholipids by the Cfa enzyme. Cyclopropane fatty acids have been demonstrated as very important to membrane permeability to protons and thus to survival in acidic conditions (Chang and Cronan, 1999; Shabala and Ross, 2008). However, engineering of *S. cerevisiae* to contain up to 10% (by area) C17cyc in the membrane was also not helpful for carboxylic acid tolerance (Liu et al., 2013b). The results support the proposition that it is transport of the anion and neutral forms of the carboxylic acid, and not the proton, that is problematic for microbial growth.

The cell membrane contains more than just phospholipids. Alterations in the abundance or structure of other membrane components can also impact carboxylic acid sensitivity. Disruption of ergosterol content in *S. cerevisiae* membranes via deletion of *erg4* increased sensitivity to undecanoic (C11:0), 10-undecanoic (C11:1Δ10) and dodecanoic acids (McDonough et al., 2002). Note that ergosterol is 22-carbon sterol that typically accounts for more than 60% of the *S. cerevisiae* sterol content (McDonough et al., 2002). We were unable to identify any reports of attempts to increase carboxylic acid tolerance via increases of ergosterol content, though it has been shown that exogenous ergosterol supplementation increases tolerance to the cyclic terpene hydrocarbon limonene (Liu et al., 2013a).

The lipopolysaccharide leaflet on the outer membrane provides a substantial barrier to diffusion; the diffusion of hydrophobic steroid probes was shown to be two orders of magnitude slower through this leaflet than through model phospholipid bilayer membranes (Nikaido, 2003). However, this means that mutations or defects that disrupt this leaflet, resulting in the “deep rough” phenotype enable increased vulnerability to compounds that enter the cell primarily through diffusion (Nikaido, 2003). Changes in the lipopolysaccharide structure, such as deletion of certain side chains, can also increase weak acid sensitivity (Barua et al., 2002; Nikaido, 2003). This is a tantalizing area of focus for engineering tolerance to carboxylic acids.

## INTRACELLULAR ANION ACCUMULATION

Transport of carboxylic acids into the cell interior, and presumably their accumulation during production, can have a variety of effects on cellular processes. Acidification of the cell interior has been recognized as a key effect of carboxylic acids (Ricke, 2003). This acidification can occur, for example, when the non-ionic HA form enters the cell and then dissociates into H^+^ and A^-^ ions.

Treatment with 56 mg/L (0.39 mM) octanoic acid resulted in a drop of intracellular pH to below 5.5 for approximately 80% of *S. cerevisiae* cells at 30°C, pH 4.0 (Viegas et al., 1998). Contrastingly, only 30% of cells had an intracellular pH below 5.5 in the control condition. In addition to the potential inhibition of enzymatic processes at this low pH, this acidification imposes a metabolic burden through the use of the ATP-dependent ATPase enzyme to remove the excess protons (Viegas et al., 1998). Studies of the relationship between temperature, carboxylic acid toxicity and intracellular pH noted that toxicity varied with temperature while intracellular pH did not, and thus the authors concluded that toxicity is not totally explained by the decrease in intracellular pH (Viegas and Sa-Correia, 1995). Transcriptome analysis of carboxylic acid-producing *E. coli* strains showed evidence of acid stress when analyzed in shake flask cultures, but not during growth in controlled fermentors (Lennen et al., 2011). This difference could possibly be due to differences in oxygen availability, as oxidative phosphorylation may be needed in order to produce the ATP needed for proton export, and other stress response components.

In addition to burdens imposed by excess protons, carboxylic anions can accumulate to high concentrations, an effect that has mainly been studied in regards to acetic acid. For example, *E. coli* K12 strains accumulated up to 30 g/L (500 mM) internal acetate during challenge with 4.8 g/L (80 mM) exogenous acetic acid (DiezGonzalez and Russell, 1997). Other possibly problems associated with anion accumulation include changes in cell turgor (Comte et al., 2007) and disruption of key amino acid pools (Roe et al., 2002).

Just as there are possible changes in cell membrane properties that can increase resistance to carboxylic acids, there are changes that can help mitigate anion accumulation. Specifically, it has been shown that when challenged with 4.8 g/L (80 mM) acetate at pH 5.9, the K12 *E. coli* strain maintains an intracellular pH of 6.8 and accumulates up to 30 g/L (500 mM) intracellular acetate. Contrastingly, the acid-tolerant *E. coli* O157:H7 maintains an intracellular pH of 6.1 and accumulates only 18 g/L (300 mM) intracellular acetate even when external acetate concentrations are as high as 9.6 g/L (160 mM) (DiezGonzalez and Russell, 1997). The authors of this study concluded that this ability to withstand and maintain a lower intracellular pH, as well as the production of excess D-lactate, decreased the driving force for carboxylic acid transport and thus the magnitude of anion accumulation (DiezGonzalez and Russell, 1997), consistent with the conclusion of an earlier study (Russell, 1992). The genetic elements driving these differences remain unclear, but are attractive targets for future engineering efforts. Insight provided by extensive studies of *E. coli* survival in acidic conditions may provide insight (Foster, 2004).

## OTHER EFFECTS

Many transcriptome-based studies have noted activation of oxidative stress response genes during production of or challenge with various carboxylic acids (King et al., 2010; Legras et al., 2010; Lennen et al., 2011; Ruenwai et al., 2011). Further tests in *S. cerevisiae* have confirmed not only increased production of reactive oxygen species in these conditions (Teixeira et al., 2004; Cipak et al., 2008; Ruenwai et al., 2011), but also increased activity of ROS-scavenging catalase and superoxide dismutase enzymes (Teixeira et al., 2004; Cipak et al., 2008). This increased abundance of ROS in yeast has largely been attributed to damage of the cell membrane and/or damage to the mitochondrial membrane (Mollapour et al., 2008; King et al., 2010; Lennen et al., 2011; Ruenwai et al., 2011). Damage to the mitochondrial membrane not only results in decreased function of the mitochondrial respiratory chain, but can also result in mutagenesis of the vulnerable mitochondrial DNA (Piper, 1999).

There are presumably other metabolic problems imposed by carboxylic acids, both in their neutral and ionic forms, that accumulate during exogenous challenge or production. The bulk of studies at this time have focused on the most apparent targets: the cell membrane, anion accumulation and cytoplasm acidification. However, there are hints of other effects. For example, it has been suggested that butyrate (C4:0) interacts directly with the leucine-responsive Lrp molecule (Nakanishi et al., 2009; Tobe et al., 2011), presumably due to structural similarities between butyrate and leucine. Thus far, these studies have mainly been motivated by the role of butyrate in regulating virulence. However, Lrp is a global regulator of *E. coli* metabolism (Yokoyama et al., 2006) and this interaction, if it extends to longer-chain carboxylic acids, could have a significant impact on the metabolism of producer strains.

Accumulation of carboxylate anions could increase the ionic strength of the cell interior, potentially inhibiting the activity of enzymes such as homocysteine transmethylase (MetE; Whitfield et al., 1970), an enzyme required for methionine biosynthesis. Intriguingly, accumulation of the MetE precursor homocysteine, which is itself toxic, has been shown to occur during acetate-mediated growth inhibition (Roe et al., 2002). This inhibition of MetE, and other ionic strength- or pH-sensitive enzymes, may have long-reaching effects on the biocatalyst metabolism.

Problems such as these may not yet be apparent due to “masking” by responses to membrane damage and acid stress. Once these primary problems are addressed, other metabolic problems may be detected.

## CONCLUSION

Carboxylic acids are a tantalizing class of biorenewable chemicals, but it appears that their toxicity is currently limiting further advances in biocatalyst performance. Toxicity is largely related to membrane damage, but additional metabolic effects warrant further investigation. However, the assertion that addressing toxicity could improve biocatalyst performance is tempered by Lennen and Pfeger’s (2013) finding that metabolic engineering strategies that increased carboxylic acid tolerance did not result in increased carboxylic acid titers. It is not yet clear if other strategies to improve robustness to these compounds will actually enable improved biocatalyst performance in terms of yields, titers and productivities.

**FIGURE 1:**
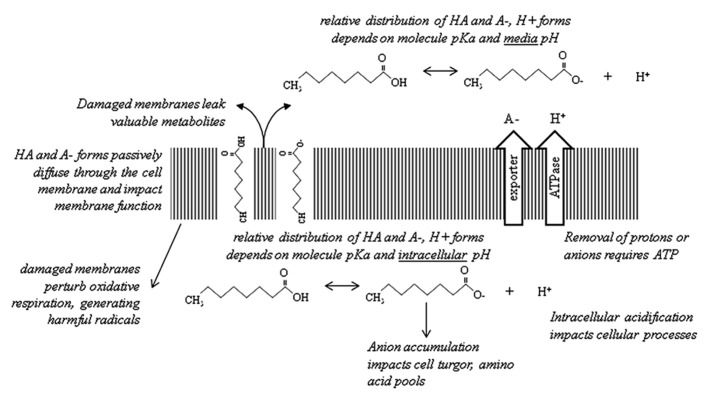
**Summary of the major mechanisms of carboxylic acid toxicity discussed in this article.** For simplicity, the cell wall/membrane(s) are shown as a single structure. Octanoic acid (C8) is used as a representative carboxylic acid.

Here we have briefly reviewed the current knowledge regarding carboxylic acid toxicity (**Figure 1**) and attempts to increase tolerance. A recent review of *E. coli*-based carboxylic acid production proposed engineering of carboxylic acid exporters and regulation of membrane composition as two key areas for future study (Lennen and Pfleger, 2012). We agree wholeheartedly with these suggestions. Most pressing is the need to address membrane damage; such work would be beneficial for solvent tolerance as well. It is not yet clear if intracellular acidification is a problem for carboxylic acid-producing strains. Other metabolic burdens associated with production of these compounds should become apparent as currently-known problems are addressed.

## Conflict of Interest Statement

The authors declare that the research was conducted in the absence of any commercial or financial relationships that could be construed as a potential conflict of interest.

## References

[B1] Abbott D. A. Knijnenburg T. A. de Poorter L. M. I. Reinders M. J. T. Pronk J. T van Maris A. J. A. (2007). Generic and specific transcriptional responses to different weak organic acids in anaerobic chemostat cultures of *S. cerevisiae*. *FEMS Yeast Res.* 7 819–833 10.1111/j.1567-1364.2007.00242.x 17484738

[B2] Abbott D. A. Zelle R. M. Pronk J. T van Maris A. J. A. (2009). Metabolic engineering of *S. cerevisiae* for production of carboxylic acids: current status and challenges. *FEMS Yeast Res.* 9 1123–1136 10.1111/j.1567-1364.2009.00537.x 19566685

[B3] Al-Awqati Q. (1999). One hundred years of membrane permeability: does Overton still rule? *Nat. Cell Biol.* 1 E201–E202 10.1038/70230 10587658

[B4] Aono R. Kobayashi H. (1997). Cell surface properties of organic solvent-tolerant mutants of *Escherichia coli* K-12. *Appl. Environ. Microbiol.* 63 3637–3642 9293016 10.1128/aem.63.9.3637-3642.1997 PMC168671

[B5] Barua S. Yamashino T. Hasegawa T. Yokoyama K. Torii K. Ohta M. (2002). Involvement of surface polysaccharides in the organic acid resistance of Shiga Toxin-producing *Escherichia coli* O157:H7. *Mol. Microbiol.* 43 629–640 10.1046/j.1365-2958.2002.02768.x 11929520

[B6] Cabral M. G. Viegas C. A. Sa-Correia I. (2001). Mechanisms underlying the acquisition of resistance to octanoic-acid-induced-death following exposure of *S. cerevisiae* to mild stress imposed by octanoic acid or ethanol. *Arch. Microbiol.* 175 301–307 10.1007/s002030100269 11382226

[B7] Carlos Serrano-Ruiz J. Pineda A. Mariana Balu A. Luque R. Manuel Campelo J. Angel Romero A. (2012). Catalytic transformations of biomass-derived acids into advanced biofuels. *Catal. Today * 195 162–168 10.1016/j.cattod.2012.01.009

[B8] Carpenter C. E. Broadbent J. R. (2009). External concentration of organic acid anions and pH: key independent variables for studying how organic acids inhibit growth of bacteria in mildly acidic foods. *J. Food Sci.* 74 R12–R15 10.1111/j.1750-3841.2008.00994.x 19200113

[B9] Chang Y. Y. Cronan J. E. (1999). Membrane cyclopropane fatty acid content is a major factor in acid resistance of *Escherichia coli*. *Mol. Microbiol.* 33 249–259 10.1046/j.1365-2958.1999.01456.x 10411742

[B10] Chotani G. Dodge T. Hsu A. Kumar M. LaDuca R. Trimbur D. (2000). The commercial production of chemicals using pathway engineering. * Biochim. Biophys. Acta* 1543 434–455 10.1016/S0167-4838(00)00234-X 11150618

[B11] Cipak A. Jaganjac M. Tehlivets O. Kohlwein S. D. Zarkovic N. (2008). Adaptation to oxidative stress induced by polyunsaturated fatty acids in yeast. * Biochim. Biophys. Acta* 1781 283–287 10.1016/j.bbalip.2008.03.010 18452720

[B12] Comte K. Holland D. P. Walsby A. E. (2007). Changes in cell turgor pressure related to uptake of solutes by *Microcystis* sp. strain 8401. *FEMS Microbiol. Ecol.* 61 399–405 10.1111/j.1574-6941.2007.00356.x 17623025

[B13] Cummings J. H. Macfarlane G. T. (1991). The control and consequences of bacterial fermentation in the human colon. *J. Appl. Bacteriol.* 70 443–459 10.1111/j.1365-2672.1991.tb02739.x 1938669

[B14] Desbois A. P. Smith V. J. (2010). Antibacterial free fatty acids: activities, mechanisms of action and biotechnological potential. *Appl. Microbiol. Biotechnol.* 85 1629–1642 10.1007/s00253-009-2355-3 19956944

[B15] DiezGonzalez F. Russell J. B. (1997). The ability of *Escherichia coli* O157:H7 to decrease its intracellular pH and resist the toxicity of acetic acid. *Microbiology* 143 1175–1180 10.1099/00221287-143-4-1175 9141680

[B16] Dunlop M. J. Dossani Z. Y. Szmidt H. L. Chu H. C. Lee T. S. Keasling J. D. (2011). Engineering microbial biofuel tolerance and export using efflux pumps. *Mol. Syst. Biol.* 7 487 10.1038/msb.2011.21 21556065 PMC3130554

[B17] Evtodienko V. Y. Kovbasnjuk O. N. Antonenko Y. N. Yaguzhinsky L. S. (1996). Effect of the alkyl chain length of monocarboxylic acid on the permeation through bilayer lipid membranes. *Biochim. Biophys. Acta* 1281 245–251 10.1016/0005-2736(96)00023-5 8664324

[B18] Foster J. W. (2004). *Escherichia coli* acid resistance: tales of an amateur acidophile. *Nat. Rev. Microbiol.* 2 898–907 10.1038/nrmicro1021 15494746

[B19] Jarboe L. R. Liu P. Royce L. A. (2011). Engineering inhibitor tolerance for the production of biorenewable fuels and chemicals. *Curr. Opin. Chem. Eng.* 1 38–42 10.1016/j.coche.2011.08.003

[B20] Jarboe L. R. Zhang X. Wang X. Moore J. C. Shanmugam K. T. Ingram L. O. (2010). Metabolic engineering for production of biorenewable fuels and chemicals: contributions of synthetic biology. *J. Biomed. Biotechnol.* 2010 761042 10.1155/2010/761042 PMC2857869 20414363

[B21] Kabara J. J. Marshall D. L. (2010). “Medium-chain fatty acids and esters,” in *Antimicrobials in Food* 3rd Edn eds Davidson P. M. Sofos J. N. Branen A. L. (Boca Raton: CRC Press) 327–360

[B22] Kabara J. J. Swieczko D. M. Truant J. P. Conley A. J. (1972). Fatty acids and derivatives as antimicrobial agents. *Antimicrob. Agents Chemother.* 2 23–28 10.1128/AAC.2.1.23 4670656 PMC444260

[B23] Kamp F. Hamilton J. A. (2006). How fatty acids of different chain length enter and leave cells by free diffusion. *Prostaglandins Leukot. Essent. Fatty Acids* 75 149–159 10.1016/j.plefa.2006.05.003 16829065

[B24] Kasemets K. Kahru A. Laht T.-M. Paalme T. (2006). Study of the toxic effect of short- and medium-chain monocarboxylic acids on the growth of *S. cerevisiae* using the CO2-auxo-accelerostat fermentation system. *Int. J. Food Microbiol.* 111 206–215 10.1016/j.ijfoodmicro.2006.06.002 16945441

[B25] King T. Lucchini S. Hinton J. C. D. Gobius K. (2010). Transcriptomic analysis of *Escherichia coli* O157:H7 and K-12 cultures exposed to inorganic and organic acids in stationary phase reveals acidulant- and strain-specific acid tolerance responses. *Appl. Environ. Microbiol.* 76 6514–6528 10.1128/AEM.02392-09 20709847 PMC2950450

[B26] Legras J. L. Erny C. Le Jeune C. Lollier M. Adolphe Y. Demuyter C. (2010). Activation of two different resistance mechanisms in *S. cerevisiae* upon exposure to octanoic and decanoic acids. *Appl. Environ. Microbiol.* 76 7526–7535 10.1128/AEM.01280-10 20851956 PMC2976208

[B27] Lennen R. M. Braden D. J. West R. M. Dumesic J. A. Pfleger B. F. (2010). A process for microbial hydrocarbon synthesis: overproduction of fatty acids in *Escherichia coli* and catalytic conversion to alkanes. *Biotechnol. Bioeng.* 106 193–202 10.1002/bit.22660 20073090 PMC3833807

[B28] Lennen R. M. Kruziki M. A. Kumar K. Zinkel R. A. Burnum K. E. Lipton M. S. (2011). Membrane stresses induced by overproduction of free fatty acids in *Escherichia coli*. *Appl. Environ. Microbiol.* 77 8114–8128 10.1128/AEM.05421-11 21948837 PMC3208990

[B29] Lennen R. M. Pfleger B. F. (2012). Engineering *Escherichia coli* to synthesize free fatty acids. *Trends Biotechnol.* 30 659–667 10.1016/j.tibtech.2012.09.006 23102412 PMC3856887

[B30] Lennen R. M. Pfleger B. F. (2013). Modulating membrane composition alters free fatty acid tolerance in *Escherichia coli*. *PLoS ONE* 8:e54031 10.1371/journal.pone.0054031 PMC3549993 23349781

[B31] Liu J. Zhu Y. Du G. Zhou J. Chen J. (2013a). Exogenous ergosterol protects *S. cerevisiae* from d-limonene stress. *J. Appl. Microbiol.* 114 482–491 10.1111/jam.12046 23082823

[B32] Liu P. Chernyshov A. Najdi T. Fu Y. Dickerson J. Sandmeyer S. (2013b). Membrane stress caused by octanoic acid in *S. cerevisiae*. *Appl. Microbiol. Biotechnol.* 97 3239–3251 10.1007/s00253-013-4773-5 23435986

[B33] Liu P. Jarboe L. R. (2012). Metabolic engineering of biocatalysts for carboxylic acids production. *Comput. Struct. Biotechnol.* 3 e201210011 10.5936/csbj.201210011 PMC3962109 24688671

[B34] Luo L. H. Seo P.-S. Seo J.-W. Heo S.-Y. Kim D.-H. Kim C. H. (2009). Improved ethanol tolerance in *Escherichia coli* by changing the cellular fatty acids composition through genetic manipulation. *Biotechnol. Lett.* 31 1867–1871 10.1007/s10529-009-0092-4 19685209

[B35] Mäki-Arvela P. Kubickova I. Snare M. Eränen K. Murzin D. Y. (2007). Catalytic deoxygenation of fatty acids and their derivatives. *Energy Fuels.* 21 30–41 10.1021/ef060455v

[B36] Marguet D. Lenne P.-F. Rigneault H. He H.-T. (2006). Dynamics in the plasma membrane: how to combine fluidity and order. *EMBO J.* 25 3446–3457 10.1038/sj.emboj.7601204 16900097 PMC1538569

[B37] Marounek M. Skrivanova E. Rada V. (2003). Susceptibility of *Escherichia coli* to C-2-C-18 fatty acids. *Folia Microbiol.* 48 731–735 10.1007/BF02931506 15058184

[B38] McDonough V. Stukey J. Cavanagh T. (2002). Mutations in erg4 affect the sensitivity of *S. cerevisiae* to medium-chain fatty acids. *Biochim. Biophys. Acta* 1581 109–118 10.1016/S1388-1981(02)00127-0 12020638

[B39] Mills T. Y. Sandoval N. R. Gill R. T. (2009). Cellulosic hydrolysate toxicity and tolerance mechanisms in *Escherichia coli*. *Biotechnol. Biofuels* 2 26 10.1186/1754-6834-2-26 PMC2770041 19832972

[B40] Mollapour M. Shepherd A. Piper P. W. (2008). Novel stress responses facilitate *S. cerevisiae* growth in the presence of the monocarboxylate preservatives. *Yeast* 25 169–177 10.1002/yea.1576 18240334

[B41] Nakanishi N. Tashiro K. Kuhara S. Hayashi T. Sugimoto N. Tobe T. (2009). Regulation of virulence by butyrate sensing in enterohaemorrhagic *Escherichia coli*. *Microbiology* 155 521–530 10.1099/mic.0.023499-0 19202100

[B42] Nikaido H. (2003). Molecular basis of bacterial outer membrane permeability revisited. *Microbiol. Mol. Biol. Rev.* 67 593–656 10.1128/MMBR.67.4.593-656.2003 14665678 PMC309051

[B43] Park J. H. Jang Y.-S. Lee J. W. Lee S. Y. (2011). *Escherichia coli* W as a new platform strain for the enhanced production of L-valine by systems metabolic engineering. *Biotechnol. Bioeng.* 108 1140–1147 10.1002/bit.23044 21191998

[B44] Perez J. M. Richter H. Loftus S. E. Angenent L. T. (2013). Biocatalytic reduction of short-chain carboxylic acids into their corresponding alcohols with syngas fermentation. *Biotechnol. Bioeng.* 110 1066–1077 10.1002/bit.24786 23172270

[B45] Piper P. Mahe Y. Thompson S. Pandjaitan R. Holyoak C. Egner R. (1998). The Pdr12 ABC transporter is required for the development of weak organic acid resistance in yeast. *EMBO J.* 17 4257–4265 10.1093/emboj/17.15.4257 9687494 PMC1170759

[B46] Piper P. W. (1999). Yeast superoxide dismutase mutants reveal a pro-oxidant action of weak organic acid food preservatives. *Free Radic. Biol. Med.* 27 1219–1227 10.1016/S0891-5849(99)00147-1 10641714

[B47] Rakowska P. D. Jiang H. Ray S. Pyne A. Lamarra B. Carr M. (2013). Nanoscale imaging reveals laterally expanding antimicrobial pores in lipid bilayers. *Proc. Natl. Acad. Sci. U.S.A.* 110 8918–8923 10.1073/pnas.1222824110 23671080 PMC3670350

[B48] Ramos J. L. Duque E. Gallegos M. T. Godoy P. Ramos-Gonzalez M. I. Rojas A. (2002). Mechanisms of solvent tolerance in gram-negative bacteria. *Annu. Rev. Microbiol.* 56 743–768 10.1146/annurev.micro.56.012302.161038 12142492

[B49] Ranganathan S. Tee T. W. Chowdhury A. Zomorrodi A. R. Yoon J. M. Fu Y. (2012). An integrated computational and experimental study for overproducing fatty acids in *Escherichia coli*. *Metab. Eng.* 14 687–704 10.1016/j.ymben.2012.08.008 23036703

[B50] Ricke S. C. (2003). Perspectives on the use of organic acids and short chain fatty acids as antimicrobials. *Poult. Sci.* 82 632–639 12710485 10.1093/ps/82.4.632

[B51] Roe A. J. O’Byrne C. McLaggan D. Booth I. R. (2002). Inhibition of *Escherichia coli* growth by acetic acid: a problem with methionine biosynthesis and homocysteine toxicity. *Microbiology* 148 2215–2222 12101308 10.1099/00221287-148-7-2215

[B52] Rossi G. Sauer M. Porro D. Branduardi P. (2010). Effect of HXT1 and HXT7 hexose transporter overexpression on wild-type and lactic acid producing *S. cerevisiae* cells. *Microb. Cell Fact.* 9 15 10.1186/1475-2859-9-15 PMC2848207 20214823

[B53] Rothen S. A. Sauer M. Sonnleitner B. Witholt B. (1998). Biotransformation of octane by *E-coli* HB101[pGEc47] on defined medium: octanoate production and product inhibition. *Biotechnol. Bioeng.* 58 356–365 10.1002/(SICI)1097-0290(19980520)58:4<356::AID-BIT2>3.0.CO;2-I 10099269

[B54] Royce L. A. Liu P. Stebbins M. Hanson B. C. Jarboe L. (2013). The damaging effects of short chain fatty acids on *Escherichia coli* membranes. *Appl. Microbiol. Biotechnol.* 10.1007/s00253-013-5113-5 [Epub ahead of print] PMC3757260 23912117

[B55] Ruenwai R. Neiss A. Laoteng K. Vongsangnak W. Dalfard A. B. Cheevadhanarak S. (2011). Heterologous production of polyunsaturated fatty acids in *S. cerevisiae* causes a global transcriptional response resulting in reduced proteasomal activity and increased oxidative stress. *Biotechnol. J.* 6 343–356 10.1002/biot.201000316 21184438

[B56] Ruffing A. M Jones H. D. T. (2012). Physiological effects of free fatty acid production in genetically engineered *Synechococcus elongatus* PCC 7942. *Biotechnol. Bioeng.* 109 2190–2199 10.1002/bit.24509 22473793 PMC3428126

[B57] Russell A. D. (1991). Mechanisms of bacterial resistance to non-antibiotics – food-additives and food and pharmaceutical preservatives. *J. Appl. Bacteriol.* 71 191–201 10.1111/j.1365-2672.1991.tb04447.x 1955413

[B58] Russell J. B. (1992). Another explanation for the toxicity of fermentation acids at low pH – anion accumulation versus uncoupling. *J. Appl. Bacteriol.* 73 363–370 10.1111/j.1365-2672.1992.tb04990.x

[B59] Segura A. Molina L. Fillet S. Krell T. Bernal P. Munoz-Rojas J. (2012). Solvent tolerance in Gram-negative bacteria. *Curr. Opin. Biotechnol.* 23 415–421 10.1016/j.copbio.2011.11.015 22155018

[B60] Shabala L. Ross T. (2008). Cyclopropane fatty acids improve *Escherichia coli* survival in acidified minimal media by reducing membrane permeability to H^+^ and enhanced ability to extrude H^+^. *Res. Microbiol.* 159 458–461 10.1016/j.resmic.2008.04.011 18562182

[B61] Shanks B. H. (2010). Conversion of biorenewable feedstocks: new challenges in heterogeneous catalysis. *Indust. Eng. Chem. Res.* 49 10212–10217 10.1021/ie100487r

[B62] Stratford M. Anslow P. A. (1996). Comparison of the inhibitory action on *S. cerevisiae* of weak-acid preservatives, uncouplers, and medium-chain fatty acids. *FEMS Microbiol. Lett.* 142 53–58 10.1111/j.1574-6968.1996.tb08407.x 8759790

[B63] Teixeira M. C. Telo J. P. Duarte N. F. Sa-Correia I. (2004). The herbicide 2,4-dichlorophenoxyacetic acid induces the generation of free-radicals and associated oxidative stress responses in yeast. *Biochem. Biophys. Res. Commun.* 324 1101–1107 10.1016/j.bbrc.2004.09.158 15485668

[B64] Tenreiro S. Nunes P. A. Viegas C. A. Neves M. S. Teixeira M. C. Cabral M. G. (2002). AQR1 gene (ORF YNL065w) encodes a plasma membrane transporter of the major facilitator superfamily that confers resistance to short-chain monocarboxylic acids and quinidine in S. cerevisiae. *Biochem. Biophys. Res. Commun.* 292 741–748 10.1006/bbrc.2002.6703 11922628

[B65] Tobe T. Nakanishi N. Sugimoto N. (2011). Activation of motility by sensing short-chain fatty acids via two steps in a flagellar gene regulatory cascade in enterohemorrhagic *Escherichia coli*. *Infect. Immun.* 79 1016–1024 10.1128/IAI.00927-10 21149585 PMC3067497

[B66] Viegas C. A. Almeida P. F. Cavaco M. Sa-Correia I. (1998). The H^+^-ATPase in the plasma membrane of *S. cerevisiae* is activated during growth latency in octanoic acid-supplemented medium accompanying the decrease in intracellular pH and cell viability. *Appl. Environ. Microbiol.* 64 779–783 9464423 10.1128/aem.64.2.779-783.1998 PMC106119

[B67] Viegas C. A. Sa-Correia I. (1995). Toxicity of octanoic-acid in *S. cerevisiae* at temperatures between 8.5 and 30°C. *Enz. Microb. Technol.* 17 826–831 10.1016/0141-0229(94)00111-4

[B68] Walter A. Gutknecht J. (1984). Monocarboxylic acid permeation through lipid bilayer membranes. *J. Membr. Biol.* 77 255–264 10.1007/BF01870573 6699907

[B69] Wang H.-H. Zhou X.-R. Liu Q. Chen G.-Q. (2011). Biosynthesis of polyhydroxyalkanoate homopolymers by *Pseudomonas putida*. *Appl. Microbiol. Biotechnol.* 89 1497–1507 10.1007/s00253-010-2964-x 21046374

[B70] Wang X. Yomano L. P. Lee J. Y. York S. W. Zheng H. Mullinnix M. T. (2013). Engineering furfural tolerance in *Escherichia coli* improves the fermentation of lignocellulosic sugars into renewable chemicals. *Proc. Natl. Acad. Sci. U.S.A.* 110 4021–4026 10.1073/pnas.1217958110 23431191 PMC3593909

[B71] Whitfield C. Steers E. J. Weissbach H. (1970). Purification and properties of 5-methyltetrahydropteroyltriglutamate-homocysteine transmethylase. *J. Biol. Chem.* 245 390–401 4904482

[B72] Yokoyama K. Ishijima S. A. Clowney L. Koike H. Aramaki H. Tanaka C. (2006). Feast/famine regulatory proteins (FFRPs): *Escherichia coli* Lrp, AsnC and related archaeal transcription factors. *FEMS Microbiol. Rev.* 30 89–108 10.1111/j.1574-6976.2005.00005.x 16438681

[B73] Zaldivar J. Ingram L. O. (1999). Effect of organic acids on the growth and fermentation of ethanologenic *Escherichia coli* LY01. *Biotechnol. Bioeng.* 66 203–210 10.1002/(SICI)1097-0290(1999)66:4<203::AID-BIT1>3.0.CO;2-# 10578090

[B74] Zhang X. Agrawal A. San K.-Y. (2012a). Improving fatty acid production in *Escherichia coli* through the overexpression of malonyl coA-Acyl carrier protein transacylase. *Biotechnol. Prog.* 28 60–65 10.1002/btpr.716 22038854

[B75] Zhang X. Li M. Agrawal A. San K.-Y. (2012b). Efficient free fatty acid production in *Escherichia coli* using plant acyl-ACP thioesterases. *Metab. Eng.* 13 713–722 10.1016/j.ymben.2011.09.007 22001432

